# Aging Into Precarity: Poverty and Health Insurance Coverage Among Older Unauthorized Latino Immigrants

**DOI:** 10.1093/geroni/igaf122.1868

**Published:** 2025-12-31

**Authors:** Mara Sheftel, Ashton Verdery, Jennifer Van Hook, James Bachmeier, Alexis Santos

**Affiliations:** Rutgers University, New Brunswick, New Jersey, United States; The Pennsylvania State University, University Park, Pennsylvania, United States; The Pennsylvania State University, University Park, Pennsylvania, United States; Temple University, Philadelphia, Pennsylvania, United States; The Pennsylvania State University, University Park, Pennsylvania, United States

## Abstract

The US immigrant population, including those who are unauthorized, is quickly aging in place. Ineligible for Social Security and Medicare, unauthorized immigrants may age at increased risk of poverty and uninsurance. Due to data limitations, the scale of their potential disadvantage remains unknown. We pioneer and validate an innovative approach to infer legal status for foreign-born respondents in the Health and Retirement Study (HRS), based on linkage to Social Security administrative records. Using this method, we find that by age 76, 56% of unauthorized foreign-born Latinos are expected to be in poverty and 59% uninsured, rates that are considerably higher than their authorized foreign-born peers, for whom 30% are estimated to be in poverty and 4% uninsured. We combine these estimates with high quality projections to examine their population-level implications. Projection results show that 20% of the growth in older adult poverty (1.1 million) and 64% of the growth in older adult uninsurance rates (1.3 million) are attributable to increases in the unauthorized population. This is notable considering the unauthorized population contributes only 6% of the growth in the total population age 65 and older in the next 20 years. Our findings provide striking evidence that legal status is a critical source of stratification for older immigrants with implications for the US population as a whole. The consequences of continued exclusion from public programs will have cascading effects, potentially impacting second- and third-generation immigrants and pressuring health systems, thereby extending far beyond the health and wellbeing of older immigrants themselves.

